# What Moves Parents to Recommend Digital Mental Health Tools for Youth? Behavioral Insights and Implications

**DOI:** 10.1002/jcop.70156

**Published:** 2026-10-08

**Authors:** Juan Pablo Zapata, Alyssa N. Gorkin, Tyra Bergstrom, Aileen Kangavary, Ian Sotomayor, Erica Szkody

**Affiliations:** ^1^ Department of Medical Social Sciences, Feinberg School of Medicine Northwestern University Chicago Illinois USA; ^2^ Impact Institute, Feinberg School of Medicine Northwestern University Chicago Illinois USA; ^3^ College of Education University of South Florida Tampa Florida USA

**Keywords:** adolescent, barriers, COM‐B, facilitators, mental health, parent, treatment access

## Abstract

In this study we aimed to examine behavioral determinants influencing parents' willingness to recommend digital mental health interventions (DMHIs) to their youth, guided by the Capability, Opportunity, Motivation–Behavior (COM‐B) model. We conducted semi‐structured interviews with 22 parents of adolescents aged 13–19 years with mental health concerns. Interview transcripts were thematically analyzed using a hybrid inductive‐deductive approach. Findings were mapped onto COM‐B domains. Capability barriers included low awareness, uncertainty about SSIs, and difficulty navigating digital tools. Opportunity facilitators involved embedding SSIs in trusted settings and receiving endorsements from credible sources, though views on peer recommendations were mixed. Motivation was shaped by alignment with parental values (e.g., safety, communication) and emotional responses such as fear or skepticism related to DMHIs and privacy. Results from this study highlight the need for dissemination strategies that directly address parental concerns and priorities, including culturally relevant messaging, tailored framing, and delivery through familiar and trusted community settings.

## 1 Introduction

In 2021, the United States Surgeon General, in collaboration with the American Academy of Pediatrics and the American Academy of Child and Adolescent Psychiatry, declared youth mental health a national emergency (OSG 2021). Approximately one in five children in the United States experience mental illness each year, yet fewer than half of these youth receive the mental health services they need (Hambrick et al. 2016).

Adolescents' help‐seeking for and access to professional mental health care are shaped by both attitudinal barriers (e.g., stigma, low perceived need) and practical challenges (e.g., cost, transportation, or limited availability of services; (Anderson et al. 2017; Barrow and Thomas 2022; O'Brien et al. 2016). In response, the past two decades have seen rapid growth in evidence‐based digital mental health interventions (DMHIs) for youth (Hollis et al. 2017; Lehtimaki et al. 2021), many of which are designed to overcome barriers to traditional care. Despite this promise, DMHIs remain underutilized. Understanding this gap requires attention not only to adolescents' own willingness to use these interventions, but also to parents who often shape their access to mental health care.

Emerging research identifies parents as important gatekeepers of adolescents' mental health care, meaning they significantly influence whether and how adolescents learn about, access, and engage with care (Reardon et al. 2017). For DMHIs, this gatekeeping role may be less about formal permission and more about the conditions that make use possible. Parents can shape whether and how adolescents learn about these tools, whether they view them as appropriate, and whether they have access to the resources needed to use them, including a device or internet connection. This influence may persist even when adolescents can access DMHIs independently and without formal parental consent. Across studies, for example, adolescents have reported feeling that accessing mental health support without their parents' permission could lead them to getting in trouble (Wanniarachchi et al. 2025) or anticipate parental disapproval or discomfort (d'Halluin et al. 2023). These expectations may shape whether adolescents seek their parents' encouragement or choose to access resources on their own (Radley et al. 2024). Yet limited research has examined what shapes parents' willingness to support adolescents' access to DMHIs.

Among the diverse array of DMHI formats, single‐session interventions (SSIs), defined as brief, self‐contained programs designed to be completed in one sitting (Schleider et al. 2020), offer a particularly low‐burden and scalable approach to delivering mental health support. A growing evidence base supports their effectiveness in reducing symptoms of depression and anxiety among youth (Schleider et al. 2025), especially when offered anonymously. Their brief, single‐session format may also address some common concerns about DMHIs, including concerns about data privacy and the demands of sustained engagement (Zapata et al. 2026). Because SSIs do not require ongoing participation, they may offer a more accessible option for youth and families who are hesitant to commit to longer‐term digital interventions. Given their brevity and growing evidence base, the present study focused specifically on parents' willingness to recommend SSIs to youth. We sought to identify the barriers and facilitators that shape parental recommendations, with the goal of generating actionable insights to strengthen parental support for SSIs and, ultimately, their uptake among youth.

### 1.1 Barriers to Seeking, Accessing and Engaging With Mental Health Services

Prior research on parents' help‐seeking behaviors (i.e., the actions parents take to recognize a mental health concern, seek information about treatment options, and initiate or facilitate their child's access to care) for youth mental health services (here defined as professionally delivered care) has generally focused on two groups: parents who are open to seeking help and those who are not. Among parents who are open to care, structural barriers such as limited provider availability, transportation challenges, scheduling conflicts, and financial burdens are commonly cited and can delay treatment until symptoms escalate (Hollis et al. 2017; Platell et al. 2023; Schnyder et al. 2019). For those less open to seeking help, barriers are often attitudinal, such as difficulty recognizing their child's symptoms as serious, beliefs that mental health problems are transient or self‐inflicted, or stigma surrounding mental health treatment (Dempster et al. 2013; Teagle 2002; Yeh et al. 2004). While these attitudinal and structural barriers are well documented in traditional service settings, they do not fully map onto the context of DMHIs, which can reduce many access‐related constraints (Piers et al. 2023). Because DMHIs can be accessed remotely at any time, they remove several structural barriers central to traditional care, including the need for transportation, provider availability, and scheduling around clinic hours. These features may be particularly valuable for socioeconomically disadvantaged and marginalized youth, who often face compounding barriers to in‐person services. At the same time, DMHIs do not eliminate access‐related barriers; inconsistent internet connectivity and limited access to private devices can constrain these benefits for some youth.

Instead, recent studies suggest that the primary barrier to parents facilitating DMHI use is a lack of awareness about these tools and uncertainty about their credibility and effectiveness (Lal et al. 2017; Schnyder et al. 2019). A recent review of 30 studies examining adolescents' and parents' attitudes toward DMHIs revealed that only 11 studies assessed parents' perspectives directly, rather than relying solely on youth‐reported perceptions of parental attitudes (d'Halluin et al. 2023). Among studies that directly assessed parents' views, findings were generally limited to satisfaction with youth‐focused DMHIs. While parents tended to express openness and willingness to consider DMHIs (Stallard et al. 2010; Sweeney et al. 2017), concerns about privacy, data security, and the perceived impersonal nature of DMHIs were consistent themes (Armoiry et al. 2018). Importantly, most existing research has focused on DMHIs that supplement traditional care and include some form of clinician involvement. Far less is known about how parents perceive self‐guided DMHIs, which are designed for independent use by youth without direct provider oversight. These interventions, including mobile apps and web‐based modules, have the potential to address major gaps in care, particularly for underserved youth. However, their successful uptake may still depend on parental support, which remains underexamined in the literature.

### 1.2 DMHIs: Single‐Session Interventions

SSIs represent a promising, evidence‐based modality within the broader landscape of DMHIs. Unlike many DMHIs designed for ongoing engagement, SSIs are intentionally structured as brief, self‐contained programs delivered in a single encounter (Schleider et al. 2020). The effectiveness of SSIs is supported by a growing body of rigorous research. A meta‐analysis of 50 randomized controlled trials (Schleider and Weisz 2017), as well as a recent systematic umbrella review encompassing over 400 clinical trials (Schleider et al. 2025), demonstrate that SSIs yield meaningful reductions in symptoms of depression and anxiety, with effects sustained over 3–12 months, particularly for youth experiencing mild to moderate distress. Notably, digital SSIs are especially well‐suited to address the needs of underserved populations. In addition to eliminating common structural barriers, digital SSIs have shown equitable or similar benefits among youth who are often marginalized within traditional care systems, including youth of color (McDanal et al. 2024), LGBTQ+ youth (McDanal et al. 2022), and Spanish‐speaking youth (Shroff et al. 2023).

Despite their strong empirical support, the real‐world reach of digital SSIs remains limited. Notably, when research‐related compensation is removed, youth are 30 times less likely to engage with digital SSIs (Cohen and Schleider 2022). Although recent efforts have successfully embedded digital SSIs into social media platforms to increase accessibility and visibility (Dobias et al. 2022), uptake in these real‐world contexts still falls short of rates observed in controlled research settings. Given the central role that parents play as gatekeepers to youth mental health support, and the well‐documented factors influencing their help‐seeking behaviors, identifying strategies to increase parental willingness to facilitate youth access to SSIs may be key to expanding the reach and impact of these interventions.

### 1.3 Current Study

The aim of this study was to understand the behavioral determinants of parental facilitation of youth engagement with SSIs. In particular, we focused on how parents may play an active role in recommending or supporting their child's use of SSIs for mental health support. By identifying the factors that shape parents' willingness and ability to promote these interventions, this study seeks to generate actionable insights for increasing the real‐world uptake of SSIs among youth. We build on prior research examining parents' perceptions of self‐guided SSIs by applying a theoretical framework recognized in public health and behavioral science. Specifically, we used the Capability, Opportunity, and Motivation model of Behavior (COM‐B; Michie et al. 2011) to guide both data collection and interpretation. The COM‐B model posits that behavior change occurs when an individual has the capability (e.g., knowledge, skills, self‐efficacy), the opportunity (e.g., access, time, social support), and the motivation (e.g., perceived value, attitudes) to engage in the target behavior. In this case, the target behavior was parents recommending or facilitating their child's use of an SSI.

Given the limited research to date on parental attitudes toward SSIs, this study is exploratory in nature. However, based on existing literature regarding parents' help‐seeking behaviors for youth mental health services, we anticipated that similar behavioral determinants may influence their willingness to recommend SSIs. (Reardon et al. 2017)To guide our study, we posed the following research questions:

1. What are the perceived barriers and facilitators influencing parents' willingness to recommend or support their child's use of digital SSIs?
2. How do these behavioral determinants map onto the COM‐B model domains of capability, opportunity, and motivation?
3. What implications do these findings have for designing parent‐focused implementation strategies to increase uptake of SSIs in real‐world settings?

## 2 Methods

### 2.1 Positionality Statement

This study was conducted from a constructivist epistemological perspective, acknowledging that knowledge is co‐constructed and shaped by the context, values, and lived experiences of both researchers and participants (Daly 2007). The research team intentionally reflected on and recognized the influence of their own positionalities throughout the study's design, implementation, and analysis. The study team included two post‐baccalaureate research coordinators, two PhD students (i.e., clinical psychology doctoral student, school psychology doctoral student), two faculty members with expertise in clinical psychology and qualitative research design, and three experts in single‐session interventions and youth mental health. The team represented a broad range of personal identities and lived experiences, including members with diverse gender and sexual identities, those with lived experience of mental health challenges, individuals with disabilities, Spanish speakers, and members of various racial, ethnic, and religious backgrounds. Notably, none of the authors were parents at the time of writing this manuscript and thus reflected on their limitations to understanding the phenomenological experiences of parents and parenting.

### 2.2 Study Design

This study employed a qualitative design using thematic analysis to explore the behavioral determinants of parents' willingness to recommend digital SSIs to their adolescent children. Semi‐structured individual interviews were conducted remotely via Zoom with parents or legal guardians of youth aged 13–19 years. Individual interviews were chosen over focus groups to minimize the potential for social desirability bias or peer influence on participants' responses. This study followed the Consolidated Criteria for Reporting Qualitative Research (COREQ) guidelines to ensure transparency and methodological rigor.

### 2.3 Study Materials

The primary study material was a semi‐structured interview guide developed to assess the behavioral drivers of parents' support for SSIs. The interview guide was designed to align with the COM‐B model, with questions structured to probe three domains: (1) capability (e.g., parents' knowledge and self‐efficacy in supporting SSIs), (2) opportunity (e.g., external factors such as time, access, or social norms), and (3) motivation (e.g., perceived value, concerns, or attitudes about SSIs). The guide also included prompts to explore more general beliefs about digital mental health tools and prior experiences with mental health services. Interview questions were open‐ended, allowing participants to elaborate on their thoughts, with optional probes included to encourage clarification or deeper responses when needed. Responses ranged in length from brief affirmations to several sentences, and follow‐up questions were used flexibly by interviewers to ensure comprehensive data collection. The interview guide was pilot‐tested and reviewed by members of the research team to ensure clarity, coverage of the COM‐B domains, and cultural appropriateness (Rosario et al. 2021).

### 2.4 Procedure

All study procedures were approved by the Northwestern University Feinberg School of Medicine Institutional Review Board prior to data collection. Participants were recruited through a secure internal database, which included contact information for over 300 families. To be eligible, participants had to be the parent or legal guardian of a teenager aged 13–19 years, have the teen live with them some or all of the time, feel comfortable speaking and writing in English, and have reliable internet access. Eligible participants provided informed consent electronically and completed a brief demographic questionnaire along with their availability for a remote interview. A total of 22 parents completed interviews; no participants withdrew from the study. Interviews were conducted via Zoom between June and August 2024. Immediately after completing the interview, participants received an $80 Amazon electronic gift card as compensation. No follow‐up interviews or contacts occurred.

### 2.5 Data Analysis

Interview transcripts were analyzed using MAXQDA 24 software (VERBI Software 2026). A hybrid inductive‐deductive coding approach was used to develop the codebook, guided by the COM‐B model of behavior change (Michie et al. 2011) and methodological recommendations for combining data‐ and theory‐driven coding (Fereday and Muir‐Cochrane 2006). Two members of the study team led the codebook development process, which occurred over four iterative rounds. Initially, one researcher conducted an open read‐through of all transcripts and generated preliminary codes. These codes were refined collaboratively by both coders over successive rounds, with modifications made until thematic saturation was achieved and full agreement was reached. The final codebook included deductive COM‐B codes, valence codes, and inductive codes capturing dissemination strategies and promotion approaches for SSIs. Once finalized, one team member coded the full set of transcripts using the agreed‐upon codebook. A second coder then reviewed all coded transcripts using a discrepancy‐coding approach. Discrepancies in coding were resolved through collaborative discussion between the two primary coders. In instances where consensus could not be reached, a third team member, who was involved in the development of the interview guide, served as an adjudicator to facilitate resolution. Throughout the analysis, analytic memos and field observations recorded during or immediately after the interviews were integrated to contextualize responses and enrich interpretation. The research team engaged in ongoing reflexive discussions to examine how their perspectives and positionalities may have influenced the coding process and thematic development.

## 3 Results

### 3.1 Participant Characteristics

Participants included 22 parents or legal guardians of adolescents aged 13–19 years (M age = 43.94, SD = 5.52). The majority of participants self‐identified as a woman (*n* = 21; 95.5%), and one participant self‐identified as a man (*n* = 1; 4.5%); most participants were college‐educated. Participants identified their race as White, non‐Hispanic (*n* = 10; 45.5%), Black or African American (*n* = 8; 36.4%), Asian (*n* = 2; 9.1%), and American Indian/Alaska Native alone or in combination with another race (*n* = 2; 9.1%). Just over half of participants identified as a single parent (*n* = 12; 54.5%); marital status was evenly split between married (*n* = 8; 36.4%) and never married (*n* = 8; 36.4%), with the remainder divorced (*n* = 4; 18.2%) or reporting another status (*n* = 2; 9.0%). Household incomes ranged from under $20,000 to over $100,000, with the largest group reporting incomes of $20,000 to $39,000 (*n* = 7; 31.8%).

A substantial proportion of the sample reported a personal history of mental health treatment (*n* = 14; 63.6%) as well as a history of mental health service use for their adolescent (*n* = 14; 63.6%); mental health service use was assessed via a checklist that included individual counseling or therapy, prescription medication for mental health, inpatient psychiatric hospitalization, intensive outpatient therapy programs, contact with a school counselor, community support groups, crisis hotline contact, and support through religious or spiritual communities. A full demographic breakdown is provided in Table 1.

**Table 1:** Participant demographics (*N* = 22).

Variable	M (SD)
Age in Years	43.94 (5.52)
	*n* (%)
Gender	
Self‐identified as a Woman	21 (95.5)
Self‐identified as a Man	1 (4.5)
Race	
White, non‐Hispanic (includes Middle Eastern)	10 (45.5)
Black or African American	8 (36.4)
Asian (including Asian Desi and Pacific Islander)	2 (9.1)
American Indian/Alaska Native, More than one race	1 (4.5)
American Indian/Alaska Native, White, non‐Hispanic	1 (4.5)
Income	
$0–$19,000	2 (9.1)
$20,000–$39,000	7 (31.8)
$40,000–$59,000	1 (4.5)
$60,000–$79,000	5 (22.7)
$80,000–$99,000	1 (4.5)
$100,000+	6 (27.2)
Biological/Adoptive parent	
Biological parent	21 (95.5)
Adoptive parent	1 (4.5)
Single parent	
Yes	12 (54.5)
No	10 (45.5)
Marriage status	
Married	8 (36.4)
Never married	8 (36.4)
Divorced	4 (18.2)
Other	2 (9.0)
Parent – Past mental health intervention	
Yes	14 (63.6)
No	8 (36.4)
Teen – Past mental health intervention	
Yes	14 (63.6)
No	8 (36.4)

*Note: n* = number of participants in each category.

### 3.2 Qualitative Themes

The COM‐B model served as the guiding framework for organizing and interpreting the thematic findings, allowing us to identify the behavioral determinants underlying parents' willingness to recommend digital SSIs to their adolescents. A total of 254 unique code applications were mapped to COM‐B domains: Capability (*n* = 73), Opportunity (*n* = 70), and Motivation (*n* = 111). While the overall number of codes was higher due to repeated statements across interviews, we report only unique instances per participant per theme to prevent overestimating the prevalence of specific concepts.

### 3.3 Capability

Psychological Capability. Among the 22 participants, only four (18%) reported prior awareness or familiarity with SSIs before participating in the study. Several participants expressed skepticism about how a single‐session format could meaningfully support youth mental health. Even if they had known SSIs existed, many said they would have hesitated to recommend them without a clearer understanding of their purpose, evidence base, and potential impact. As one participant explained:

I would just want to make sure that [the SSIs] are valid and don't make it worse… and that they're very good at encouraging the kids to seek help around them and not just rely on these SSIs… If they're getting to that point where they feel, you know, suicidal or something like that, they need to know that, hey, I might need help, from parents, or school counselors, or mental health professionals around them.

Several parents indicated that a key factor that would support their ability to recommend SSIs was knowing how to clearly describe the intervention's benefits and level of commitment required. Some reported having previously recommended mental health resources to their teens without feeling fully confident in how to explain them. One parent shared, “If you can show me an explanation of the benefit that I can understand and feel good about, that would really give me what I would need to be able to talk to her about it.” When asked how information about SSIs could be better communicated to parents, participants emphasized the importance of using clear, non‐technical language that connects to everyday experiences. Parents suggested that jargon‐free, relatable messaging would improve their ability to talk about SSIs with their children. As one parent explained:

Not so many clinical terms, but just things that I can understand quick. Like, does your kid have frequent arguments with their friends? Do they have trouble with this or that? Having plain examples to where I can like, ‘Oh yeah, they have trouble with that.

#### 3.3.1 Physical Capability

Of the 73 unique segments coded under Capability, 40% (*n* = 29) pertained to parents' physical capability to recommend or support their teen's use of a digital SSI. Several participants referenced logistical challenges or skill‐based limitations that affected their ability to engage with digital materials. These challenges primarily reflected discomfort or lack of familiarity with digital platforms, particularly when it came to navigating unfamiliar websites or setting up web‐based tools. For example, one parent expressed a preference for printed resources, stating they would feel more comfortable with “pamphlets” or other physical how‐to guides, rather than relying solely on digital formats. In some cases, parents noted that their own limited technical skillsets made it difficult to access or recommend SSIs. One participant, referring to their recently turned 13‐year‐old, remarked that they did not feel equipped to help their child engage with a digital tool, explaining, “even downloading information from their kid's school website” was a challenge. Additionally, several parents cited time constraints and caregiving responsibilities as barriers to engaging with new tools themselves before recommending them.

### 3.4 Opportunity

#### 3.4.1 Social Opportunity

Themes related to social opportunity, including the influence of social norms, expectations, and cues emerged as important contextual factors shaping parents' willingness to recommend or support their child's use of SSIs. Across participants, 45% (*n* = 10) indicated that endorsements from others or institutions were important, 32% (*n* = 7) said endorsements would not matter, and 18% (*n* = 4) were neutral. Those who valued endorsements primarily referenced trusted sources, such as their child's pediatrician, teacher, or school counselor. Several participants noted that endorsements held greater weight when they came from recognized organizations or authoritative bodies, rather than individual professionals with whom they did not have a personal relationship. As one parent explained:

You can't just say a doctor supports this… I want a board of American doctors. Or approved by the American Psychological Society, Psychiatrist Society, a New York City school vendor. Those types are respected because they have value…

Participants were relatively divided in how much influence other parents would have on their willingness to recommend SSIs to their youth. Several parents indicated that peer recommendations would carry little weight, particularly if the recommending parent had different values, parenting styles, or beliefs about mental health (*n* = 15; 68%). Some expressed concern that such differences could lead to distrust or even resentment among peers or parents. Others noted that conversations about youth mental health are often considered private, and that peer‐shared resources might feel inappropriate. One parent explained dismissing peer input, emphasizing social dynamics and expectations:

Never listen to other parents when you're talking about your own children. End of story. Okay? Because it's called one‐upping… If a parent tells me this, I don't want to listen. If the teacher tells me, the guidance counselor, yes. Not a parent.

Among parents who indicated that peer support would influence their willingness to recommend SSIs (*n* = 10; 45%), many expressed a desire to learn from the experiences of other families navigating similar mental health challenges. These parents noted that hearing about a specific tool that had worked for another child, particularly one they knew personally would increase their comfort in recommending it to their own teen. Several participants shared that they were already members of online parenting forums or school‐based support groups, which they used as trusted spaces for exchanging advice and resources. As one parent explained:

If a parent had a teenager who had used these and had great results, then I would be even more excited about recommending, or at least saying, ‘Hey, you might want to try this.’ So, if it's worked for one teenager, there's obviously a possibility that it'll work for another.

Some parents referenced social expectations around privacy, trust, and adolescent autonomy as key factors influencing their willingness to recommend SSIs, particularly when it came to older teens. As one parent noted, “At this age, like already close to 18, you've got to step back when you can. I think parents can be too involved, and that can be bad.” Several parents shared concerns that recommending mental health tools might be perceived by their teens as intrusive or controlling. They noted that as teens move closer to going to college, there is often a stronger desire for independence, which can make unsolicited recommendations from parents less well‐received. In turn, some parents preferred to wait until their teen initiated the conversation before offering suggestions, emphasizing a desire to respect their autonomy.

#### 3.4.2 Physical Opportunity

Of the unique 70 codes within the Opportunity domain, 34% (*n* = 24) were specific to physical opportunity, defined as the environmental cues and resources that enable behavior, and reflected factors influencing parents' willingness to recommend SSIs. Many parents discussed the importance of embedding SSIs within known settings such as schools, clinics, or pediatrician offices. These settings were viewed as convenient and accessible points of delivery that could help streamline access to the intervention while also enhancing trust, thereby overlapping with elements of social opportunity. Several parents also expressed a preference for physical, printed materials over digital formats when it came to recommending or explaining SSIs to their teens. This preference was often tied to limitations in their own comfort or familiarity with digital tools, but also reflected environmental considerations such as time constraints and the ability to follow up. As one parent stated, “…having something physical with me, would make it easier to go back and forth, because we would both forget at some point since everything else is online.”

### 3.5 Motivation

#### 3.5.1 Reflective Motivation

Of the 111 unique codes within the Motivation domain, 77% (*n* = 86) were specific to reflective motivation, focusing on parents' beliefs about the relevance and usefulness of SSIs in relation to their teen's current needs, values, and goals. Parents often evaluated whether the content covered in SSIs would meaningfully connect with their child, with many expressing greater willingness to recommend an SSI if they perceived it to be a good fit for their teen's life circumstances. The most cited area of interest among parents was content related to managing friendships and relationships, which they viewed as both developmentally appropriate and personally relevant to their adolescents. Several parents also emphasized the importance of knowing that SSIs would encourage or facilitate open communication between teens and their families. For some, this aligned closely with their own emphasis on honesty and emotional openness. As one parent explained:

Reinforcing that for us he can always come and talk to your family about it… Just letting him know that he does have support. We value being open, we value being honest because that's the only way… for us to kind of grow as a family.

Many of the additional findings within this subtheme reflected parents' deliberate beliefs and values regarding how to ensure their teen's safety when using online tools. Specifically, participants described features they believed should be integrated into SSIs to align with their expectations for responsible and secure digital engagement. Commonly desired features included transparency around privacy protections (e.g., data anonymity, who would access the information, how long data would be stored) and parental controls, such as limiting a teen's ability to exit the intervention or triggering parent notifications if certain responses indicated risk. These concerns were particularly salient when parents considered SSIs that covered more sensitive or in‐depth mental health topics. One parent shared a desire to “precheck content ahead of time” to ensure the material was appropriate and aligned with their family's safety standards. Another parent explained:

If it's just kind of subsurface level mental wellbeing, that's pretty tame, and I would feel okay. But getting into deeper severe depression topics or something like that, it might, yeah, cause some stress, so I would want to know ahead of time before sharing it with them.

#### 3.5.2 Automatic Motivation

Although fewer codes reflected automatic motivation (25 of 111), several participants described initial emotional reactions, such as discomfort, fear, or hesitation that shaped their willingness to recommend SSIs. These responses were often instinctive or gut‐level. For some parents, the terminology alone evoked uncertainty about what the intervention would entail. Others expressed discomfort with the growing role of technology in adolescents' lives, noting concerns about digital overexposure and the potential for unintended consequences. One parent remarked on the anxiety triggered by how “much stuff teens can get into with technology already.” Another participant described their initial reaction as “fear,” citing concerns about online data security. As one parent explained:

There's a lot of fear of being too honest online. Like how personal is this? You know, are they answering a survey? I just don't trust how this information can be shared, and as a parent, that makes me nervous.

## 4 Discussion

### 4.1 Overview

Given the limited prior research on parental attitudes toward SSIs, this study was exploratory. Drawing on the broader literature on parents' roles in youth mental health care, we anticipated that parents would identify capability‐related barriers, including limited knowledge of SSIs and uncertainty about appropriate parental involvement or consent, and motivation‐related facilitators specific to the SSI format, including convenience, anonymity, and its brief, low‐burden design. Our findings partially supported these expectations. Consistent with our hypotheses, most parents reported little to no prior awareness of SSIs, and this unfamiliarity appeared closely tied to the hesitation and skepticism described below. However, the anticipated motivation‐related facilitators were less central than expected. Rather than emphasizing convenience or anonymity, parents prioritized trust, transparency, and the ability to vet or control the content their adolescents would encounter. These findings suggest that parents' willingness to recommend an SSI may depend less on its efficiency or convenience than on their confidence in its credibility, safety, and fit with family values.

### 4.2 Need for Parent Awareness and Education

This study highlights that parents' decision to recommend digital mental health tools, particularly SSIs, is both complex and personal. To better understand the drivers of this behavior, we applied the COM‐B model to organize participants' perspectives across psychological and physical capability, social and physical opportunity, and both reflective and automatic motivation. A central finding was that despite the growing presence of digital technology in daily life, especially following the COVID‐19 pandemic (Panchal et al. 2023; Torous et al. 2020), which normalized online platforms for education, healthcare, and communication, parents still expressed significant uncertainty and hesitation around digital mental health tools. This hesitation was driven, in part, by a lack of awareness of SSIs, which nearly all participants reported at the start of the study. Additionally, the proliferation of digital programs with varying levels of quality and credibility created confusion and mistrust, making it more difficult for parents to evaluate and confidently recommend SSIs to their teens.

### 4.3 Parental Desire for Vetting and Collaboration Before Dissemination

Participants' concerns centered largely on the potential risks and lack of familiarity associated with digital mental health tools, exemplifying themes related to reflective motivation. Many parents expressed that they would be more willing to recommend SSIs if they had the opportunity to “vet” them in advance. Some also indicated a desire for parental control features, especially for parents navigating challenges with safe internet use with their teens. These reflections aligned with more immediate, emotion‐driven reactions parents reported when first introduced to SSIs, such as “hesitation” or unease about what the intervention might entail.

Importantly, many parents indicated they would feel more comfortable recommending SSIs if they knew the content avoided more intensive topics or, alternatively, if the programs explicitly encouraged teens to talk with their parents as part of the process. These findings underscore that trust, transparency, and perceived alignment with parenting values play a critical role in shaping both reflective and automatic motivational responses. While prior research has largely focused on youths' perceptions of whether their parents would support their use of DMHIs (d'Halluin et al. 2023), this study uniquely captures parents' own articulated motivations, concerns, and preferences. Our findings extend the existing literature by showing that although parents may initially approach SSIs with skepticism, many are open to supporting their use, particularly when interventions are seen as appropriately related, and positioned to complement family communication.

### 4.4 Importance of Trust and Institutional Credibility

Our study also builds on prior research emphasizing the importance of institutional credibility in parents' decision‐making about mental health support for their children (e.g., Reardon et al. 2017). For instance, a recent systematic review examining the barriers and facilitators to parents' willingness to seek psychological treatment for their children found that endorsement from trusted sources (Platell et al. 2023; Reardon et al. 2017), such as healthcare providers, schools, or other established institutions, consistently emerged as a key facilitator across studies. Our findings extend this literature by identifying two distinct layers of credibility that parents consider when evaluating digital health tools: (1) national‐level endorsements from well‐known professional organizations (e.g., the American Psychological Association), and (2) local‐level endorsements from trusted individuals within their local community. Parents expressed a clear preference for endorsements from these recognized sources over vague claims (e.g., “recommended by doctors”) or unfamiliar organizations lacking established credibility. Furthermore, this study is one of the first to explore parents' divergent perspectives on parent‐to‐parent recommendations, a novel dimension of social opportunity. While some parents expressed openness to hearing about SSIs from other parents, especially those in school‐based or online support groups, others viewed such interactions as intrusive or unhelpful, particularly if they perceived parenting styles or values to differ. This variation highlights the nuanced role of peer influence and suggests that future dissemination efforts should tailor messaging strategies based on both the source and perceived social proximity of the messenger (Southam‐Gerow and Prinstein 2014).

### 4.5 Preferred Dissemination Strategies

While preferred dissemination strategies targeting parents, such as tailored advertising or direct‐to‐consumer outreach remain largely underexplored in the digital mental health literature (Hollis et al. 2017), our study offers preliminary insights into how dissemination approaches might be optimized to engage parents as active facilitators of youth access to SSIs. Several participants indicated that they would be more inclined to learn about SSIs, and subsequently recommend them to their children, if they had access to printed materials such as brochures or informational handouts. These preferences also intersected with findings related to social opportunity, as parents emphasized a desire for such materials to be available in trusted, familiar settings. Notably, some parents described the utility of printed resources as a socially neutral way to introduce SSIs, particularly in cases where parent‐teen communication about mental health might be met with some resistance. For these families, having a brochure to pass along offered a less confrontational or direct method of sharing mental health support options, especially for older adolescents navigating increased autonomy.

### 4.6 Implications

Although this study did not explicitly ask parents about preferred strategies to increase their willingness to recommend SSIs, the findings point to a range of relevant Behavior Change Techniques (BCTs; Michie et al. 2011) that can be used to address barriers and enhance facilitators across domains of capability, opportunity, and motivation. Table 2 presents these theoretically grounded strategies aligned with the COM‐B model to support parents in this behavior. For example, to address physical opportunity, researchers and clinicians might consider increasing outreach via printed materials such as brochures or handouts placed in trusted environments (e.g., pediatric clinics, schools). To build psychological capability, these materials could include developmentally tailored talking points that help parents understand SSIs. Given the mixed findings regarding social influences, one recommended approach is to ensure that SSIs are introduced through locally trusted and credible sources, rather than vague or impersonal endorsements. To reduce emotional barriers and improve automatic motivation, SSIs should be framed in a neutral way, emphasizing growth, agency, and accessibility rather than pathology.

**Table 2:** Change techniques mapped to COM‐B for promoting parental recommendation of SSIs.

COM‐B domain	Barrier/facilitator	Behavior change technique	Practical application
Psychological capability	Lack of awareness about SSIs; discomfort explaining them to older teens	4.1 Instruction on how to perform the behavior	Provide clear, jargon‐free explanations and examples of how parents can introduce SSIs to their youth
	Desire to understand what SSIs are and how they work	5.1 Information about health consequences	Share concise evidence on how SSIs help with youth mental health, emphasizing real‐world outcomes
Physical capability	Limited digital literacy or confidence navigating online tools	12.5 Adding objects to the environment	Provide printed brochures, how‐to guides, and non‐digital materials in trusted spaces
	Limited time and competing caregiving responsibilities	7.1 Prompts/cues	Use simple reminders and visuals to reduce cognitive burden and make information accessible
Social opportunity	Preference for recommendations from known professionals or trusted institutions	9.1 Credible Source	Disseminate SSIs through known and respected sources (e.g., pediatricians, school counselors, APA) rather than generic “expert” endorsements
	Mixed influence of other parents	6.2 Social comparison	Where appropriate, include testimonials from relatable parents in support groups or communities
Physical opportunity	Preference for physical resources over digital‐only formats	12.1 Restructuring the physical environment	Make SSIs visible and accessible in environments parents already trust, like doctors' offices and school newsletters
	Desire for tech‐supported safety controls	12.2 Restructuring the social environment	Equip schools and clinics to help parents feel confident that SSIs are vetted
Reflective motivation	Value placed on strengthening parent‐teen relationship	5.6 Information about emotional consequences	Highlight how SSIs can promote communication and reduce distress in parent‐child dynamics
	Desire to protect children from triggering content	13.2 Framing/reframing	Position SSIs as tools for emotional resilience, early intervention, or skill‐building rather than treatment
Automatic motivation	Emotional discomfort, skepticism, or fear around mental health tools	11.2 Reduce negative emotions	Use transparent messaging about safety, privacy, and data protection to reduce fear and build trust
	Initial gut‐level resistance to the term “single‐session intervention”	13.2 Framing/reframing	Frame SSIs as “brief, guided mental health tools” or “starter strategies for emotional wellness” in communication to parents

*Note:* BCT numbers refer to the Behavior Change Technique Taxonomy v1 (Michie et al. 2013). Each entry includes an applied strategy tailored to the study context to inform future implementation and dissemination efforts.

While these recommendations require further empirical testing, future studies could experimentally evaluate the impact of different BCT “bundles” (Michie et al. 2013) on parents' willingness to recommend SSIs. These strategies are not intended to replace direct‐to‐youth dissemination efforts (Radomski et al. 2019; Schleider and Weisz 2017), but rather to complement them. Targeting both parents and adolescents concurrently may enhance implementation outcomes, especially if dissemination efforts increase parents' general knowledge and self‐efficacy regarding digital mental health tools. These factors have been consistently identified as barriers in prior research, with youth frequently reporting that their parents' lack of familiarity with such tools discourages them from seeking or discussing support (Reardon et al. 2017).

### 4.7 Limitations

The current study must be interpreted within the context of its limitations. First, the sample was composed primarily of parents who were already willing to participate in a study focused on youth mental health, and who had personally received mental health support services, which may bias the findings toward individuals with more favorable or open attitudes toward mental health interventions. As such, the perspectives of parents who hold more stigmatizing views or are less receptive to mental health support, particularly in digital formats, may be underrepresented. Additionally, the sample lacked substantial racial and ethnic diversity, limiting the generalizability of findings to more diverse parent populations. Prior research has shown that parents from different racial and ethnic backgrounds may hold distinct beliefs and preferences regarding mental health, which warrants further investigation into more representative samples. Moreover, while we employed a rigorous qualitative methodology, including the use of multiple coders, iterative codebook development, and formal assessment of thematic saturation (Guest et al. 2006), our findings are subject to inherent limitations associated with qualitative research. These include the potential for researcher interpretation bias, limited generalizability beyond the specific study context, and reliance on self‐reported perspectives, which may be shaped by social desirability or recall bias. Despite these limitations, the study offers important strengths.

## 5 Conclusion

This study highlights the complex psychological, social, and environmental factors that influence whether parents are willing to recommend SSIs to their adolescents. Our findings underscore that simply promoting the benefits of SSIs for youth is unlikely to be sufficient for increasing parental engagement. Instead, dissemination efforts must directly address parents' concerns and needs, including their desire for clear, jargon‐free information, transparency about intervention content and safety, and alignment with core parenting values, particularly open communication and trust. These strategies should be co‐developed with parents to ensure cultural and contextual relevance. By applying the COM‐B model and Behavior Change Wheel framework, this study offers theory‐driven guidance on how to reduce behavioral barriers and strengthen facilitators to parental support. These findings can inform practical behavior change techniques that enhance parents' capability, opportunity, and motivation to act as active partners in youth mental health care. To move from promise to practice, future research must test and refine these approaches in partnership with families, ensuring that digital mental health innovations are not only evidence‐based, but also accessible, trusted, and equitable.

## Author Contributions

All authors contributed to the development and writing of the manuscript. The study was conceptualized and designed by the lead author (Juan Pablo Zapata). Data analysis was led by Alyssa N. Gorkin and Aileen Kangavary, with support from Erica Szkody, Tyra Bergstrom, and Ian Sotomayor. All authors reviewed, revised, and approved the final version of the manuscript.

## Ethics Statement

All procedures performed in studies involving human participants were in accordance with the ethical standards of the institutional and/or national research committee and with the 1964 Helsinki declaration and its later amendments or comparable ethical standards.

## Conflicts of Interest

The authors declare no conflicts of interest.

## Data Availability

To protect the confidentiality and anonymity of study participants, the datasets generated and analyzed during the current study are not publicly available. However, de‐identified data may be made available from the corresponding author upon reasonable request and with appropriate institutional approvals.
